# Breast Cancer Development in a Transgender Male Receiving Testosterone Therapy

**DOI:** 10.1155/2018/3652602

**Published:** 2018-12-31

**Authors:** Nadia Barghouthi, Jennifer Turner, Jessica Perini

**Affiliations:** ^1^Department of Endocrinology, West Virginia University, Morgantown, WV, USA; ^2^Department of Medicine, West Virginia University, Morgantown, WV, USA

## Abstract

**Context:**

To describe a case of invasive ductal carcinoma of the breast in a transgender male receiving testosterone therapy for gender-affirming treatment.

**Case Description:**

A 28-year-old transgender male receiving intramuscular testosterone was found to have a breast mass on ultrasound after self-exam revealed a palpable breast lump. Ultrasound-guided breast biopsy revealed estrogen receptor/progesterone receptor (ER/PR) negative, human epidermal growth factor receptor-2 (HER-2) positive, invasive ductal carcinoma of the left breast. He underwent neoadjuvant and adjuvant chemotherapy along with bilateral mastectomy. At patient request, his testosterone injections were permanently discontinued.

**Conclusion:**

Fewer than 20 cases of breast cancer in transgender male patients have been reported in medical literature. While studies have shown increased risk of breast cancer in postmenopausal women with higher testosterone levels, data regarding premenopausal women is conflicting and little is known about breast cancer risk in transgender individuals receiving gender-affirming hormone therapy (GAHT), with inconclusive results regarding correlation between testosterone therapy and breast cancer. More research is required to evaluate whether a possible increased risk of breast cancer exists for transgender men receiving gender-affirming therapy.

## 1. Introduction

Testosterone is used in the transgender male population in order to induce masculinization; however the association between testosterone and breast cancer risk is not well defined. There are a small number of cases documenting breast cancer in transgender male patients receiving masculinizing GAHT with current studies showing a possible but inconclusive correlation between higher doses of testosterone and increased breast cancer risk.

## 2. Case Presentation

A 28-year-old transgender man who had been receiving masculinizing hormone therapy presented with a self-palpated left breast mass. Past medical history included gender incongruence for which the patient had been receiving weekly testosterone injection therapy for one year prior to presentation. Gynecological history was unremarkable and menses had stopped approximately one month into gender-affirming therapy. Home medications included intramuscular testosterone enanthate 100 mg weekly, multivitamin, and vitamin D supplement. He had never smoked and denied alcohol or illicit drug use. Family history included mother with hypertension, father with diabetes mellitus, paternal great grandmother with breast cancer, maternal great grandmother with ovarian cancer, maternal grandmother with lung cancer, and maternal grandfather with gastric cancer.

On physical exam, the patient was a well-appearing male with moderate growth of facial hair. Cardiac, pulmonary, abdominal, neurologic, and musculoskeletal exam were all unremarkable. Breast exam revealed a palpable left upper breast lump without skin dimpling or changes in pigmentation. His lab values included total testosterone ranging over the year from 544 to 970 ng/dL (reference range for men: 270-1,734), hemoglobin and hematocrit of 15.1 g/dL (reference range for men: 12.5-16.3) and 44.2% (reference range for men: 36.7-47.0), and normal hepatic function panel.

## 3. Investigation

**Figure 1:**
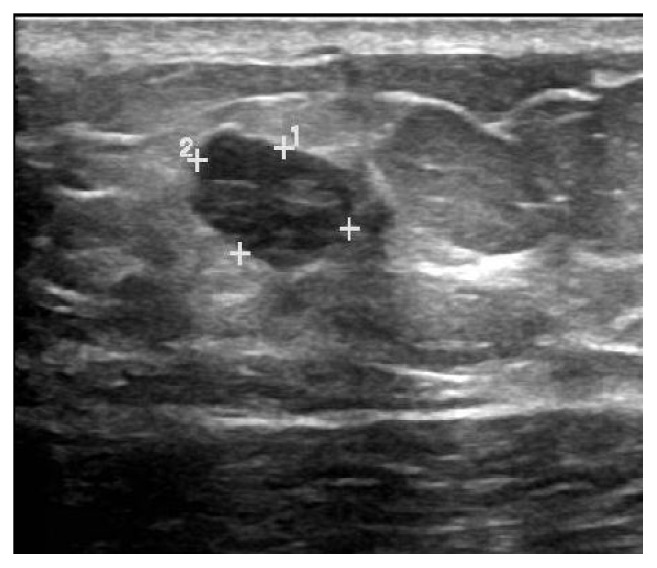
Breast ultrasound: lobulated solid left breast mass at 11:00, approximately 0.8 × 1.1 × 1.4 cm.

Breast ultrasound revealed a 1.4 × 0.8 × 1.1 cm oval, hypoechoic left upper breast mass with indistinct margins which was suspicious for malignancy and corresponded to the palpable lump on physical exam (Figure 1). Ultrasound-guided breast biopsy was performed with pathology revealing nuclear grade 3, estrogen receptor (ER)/progesterone receptor (PR) negative, human epidermal growth factor receptor 2 (HER-2) positive, invasive ductal carcinoma of left breast. Genetic testing was negative for androgen receptor (AR) mutation and breast cancer type 1 and 2 (BRCA-1 and BRCA-2) mutations but did show a variant of undetermined significance (VUS).

## 4. Treatment and Outcome

After diagnosis of invasive ductal carcinoma of the left breast, a lengthy discussion was held with the patient regarding potential risks of continuing testosterone therapy versus risks to the patient's well-being from cessation of gender-affirming hormone therapy. The patient opted to discontinue the testosterone. He underwent neo-adjuvant chemotherapy with docetaxel, carboplatin, pertuzumab, and trastuzumab. He then underwent bilateral nipple-sparing mastectomy with surgical pathology showing left breast ductal carcinoma in situ with negative margins and no lymph node involvement. The patient later had a total laparoscopic hysterectomy with bilateral salpingo-oophorectomy and remains on trastuzumab therapy at this time.

## 5. Discussion

Transgender male patients typically achieve masculinization through gender-affirming therapy with testosterone. Although there is strong evidence to link higher estrogen levels with breast cancer development [1, 2], there is mounting but inconclusive evidence suggesting a link between higher circulating androgen levels and the development of breast cancer [3–9]. The patient population in these studies has been female and, to date, there have been fewer than 20 reported cases of breast cancer related to testosterone therapy in transgender men [3–7, 10]. Two proposed mechanisms of excess androgen-related breast cancer development include aromatization of testosterone to estrogens in peripheral tissues and the activation of androgen receptors which leads to cellular growth and proliferation, particularly in mammary tissues [1–4, 8, 9]. Conversely, a few studies suggest protective effects of androgens possibly via competitive blockade of estrogen receptors in mammary epithelium [8].

Studies have noted that some breast cancers which are negative for ER and PR expression are positive for AR expression, suggesting increased androgen receptor positivity as its own risk factor in the development of breast cancers [3]. In our case, expressions of ER, PR, and AR were all negative however HER-2 expression was positive. Some case reports of breast cancer development in transgender men receiving testosterone therapy noted increased expression of HER-2 but the mechanism of testosterone-related overexpression of HER-2 is unknown [3, 11]. Of the cases revealed in the literature, the types of breast cancer were variable, consisting of invasive ductal carcinoma, neuroendocrine carcinoma, and tubular adenocarcinoma [3–7, 11]. The strongest correlation of androgen levels and risk of breast cancer was in hormone receptor positive tumors including ER/PR and androgen receptor (AR) positivity [2, 3]. One study which followed five transgender male patients two years after initiation of testosterone therapy found upregulation of over 200 genes associated with breast cancer-unique expression [3].

No consistent guidelines exist regarding the continuation of GAHT following breast cancer treatment in transgender men [3–7, 10]. Published reports suggest that most patients are restarted on low-dose testosterone therapy with or without an aromatase inhibitor to prevent peripheral conversion of testosterone to estrogens [3, 4, 6, 7, 11]; however the available evidence on the risk of breast cancer recurrence with continuation of masculinizing GAHT is conflicting. Prophylactic use of aromatase inhibitors is currently under investigation and its effectiveness is unknown [3–7]. None of the patients who resumed low-dose testosterone therapy had breast cancer recurrent at 2-5 years' follow-up in a limited number of case reports [3–7]. Other studies of recurrence among transgender men receiving masculinizing GAHT have indicated that bilateral mastectomy does not negate future risk of cancer development in residual breast tissue [4, 5]. In individual high-risk cases, such as in this case with extensive family history of cancer, pretreatment screening with breast imaging may be warranted prior to initiating GAHT. In our particular case, the patient elected to permanently discontinue testosterone therapy. Whether such cessation of therapy was necessary is uncertain and more research is required to assess benefits of therapy versus risk of breast cancer recurrence.

## Data Availability

The review of collective case report data used to support the discussion findings in this case report are included within the article within the following references [1–11].
